# Real Time 3D Echocardiographic Evaluation of Iatrogenic Atrial Septal Defects After Percutaneous Transvenous Mitral Commissurotomy

**DOI:** 10.15171/jcvtr.2015.20

**Published:** 2015

**Authors:** Sarath Babu Devarakonda, Boochi Babu Mannuva, Rajasekhar Durgaprasad, Vanajakshamma Velam, Vidya Sagar Akula, Latheef Kasala

**Affiliations:** Department of Cardiology, SVIMS, Tirupati, Andhra Pradesh, India

**Keywords:** PTMC, RT3DE, iASD

## Abstract

*Introduction:* Percutaneous transvenous mitral commissurotomy (PTMC) is a safe and effective procedure for relief of severe mitral stenosis. PTMC is being done widely and many transseptal procedures requiring large diameter catheters, sheaths are becoming popular. The knowledge of iatrogenic atrial septal defect (iASD) is vital. This study assessed the use of real-time 3D echocardiography (RT3DE) and incidence of iASD in a cohort of patients undergoing transseptal catheterization during PTMC.

*Methods:* One hundred ten patients underwent PTMC. The reliability and accuracy of RT3DE for iASD detection was determined, RT3DE was compared with 2D echocardiography (2DE) for iASD occurrence, influencing variables analyzed and followed up for 1 year.

*Results:* RT3DE is more reliable and accurate for the study of iASD than 2DE. Color RT3DE detected iASD in 94 (85.5%), with 2DE iASD was detected in 74 (67.3%) (*P * < .0001).On follow up 85% had iASD post procedure, 56% at 6 months, 19% at 1 year follow up. The mean iASD diameter was 5.41 ± 3.12 mm and area 6.57 ± 3.81 mm^2^. iASD correlated with patient height, Wilkins score, pre-PTMC LA ‘v’, and post-PTMC LVEDP.

*Conclusion:* RT3DE imaging is superior in accuracy to traditional 2DE techniques. All the modes of RT3DE are useful in the assessment of iASD. iASD measured by RT3DE correlates with several patient, procedural and echocardiographic variables.

## Introduction

Atrial septal defect (ASD) is a predestined complication of the percutaneous transvenous mitral commissurotomy (PTMC), since it requires a transseptal technique.^1,2^ The role of this iatrogenic atrial septal defect (iASD) has not been clear. They have a potential for paradoxical embolism and cerebral ischemia, they are also known to affect the accuracy of post procedure mitral valve area measurements. Their natural history is of spontaneous closure in a majority; role for any form of closure is controversial and should bear the chance of repeat transseptal procedure in the future. There also is a debate to remove iASD as a complication of PTMC from literature as it occurs potentially in all cases, many studies have shown that it is of little consequence and is very difficult to characterize. Several studies over the past decades have shown the incidence of iASD to be high, but the standard diagnostic modalities to define them including oximetry, indicator dilution, two dimensional echocardiography (2DE), color flow Doppler have been suboptimal.^3-11^

Real time 3 Dimensional echocardiography (RT3DE) already has a proven role in pre PTMC mitral valve (MV) apparatus morphology evaluation including Wilkins score assessment and is also useful in the evaluation of potential complications including mitral regurgitation and atrial septal defects. There is a potential for RT3DE guidance for difficult or high risk procedures. Currently it can be argued that RT3DE is definitely superior to 2D imaging techniques and should be routinely used in mitral stenosis, particularly in the post PTMC period, where other methods have been proven to be inaccurate.^12^

The primary aim of this study was to investigate the feasibility of imaging by various methods of transthoracic RT3DE for evaluating and quantification of iASDs in the context of PTMC in a group of consecutively imaged patients to determine the acoustic window or perspective from which the atrial septum, location of iASD, defect area are best visualized. The secondary aim was to compare 2DE and RT3DE for the evaluation of iASDs after PTMC and to study the effect of various demographic, echocardiographic and catheterization data on occurrence of iASD.

## Materials and Methods

The study population consisted of 110 patients with chronic rheumatic heart disease with symptomatic mitral stenosis (MS) who underwent PTMC in cardiology department of Sri Venkateswara Institute of Medical sciences, Tirupati, between Jun 2011 and Mar 2013. After informed consent, patients with chronic rheumatic heart disease and severe MS, in whom PTMC was feasible, were included in the study. Patients with mitral restenosis after earlier PTMC or surgical mitral commissurotomy were also included. Those with non-feasible 2D or 3D echocardiography because of poor image quality or poor echo window, patients with left atrial thrombus, mitral regurgitation greater than 2/4, calcific MV and commissures, severe aortic valve disease associated with MS, critically ill patients and pregnant were excluded.

Comprehensive transthoracic 2DE was performed the day before and 24-48 hours after PTMC using S5 probe iE33 echocardiography unit, Philips medical systems, USA as per standard guidelines.^13^ Transesophageal echocardiography (TEE) was performed with S10 probe, iE33 echocardiography unit, Philips medical systems, USA. The patients underwent PTMC by single balloon as per guidelines and institutional norms. All patients were analyzed at baseline, post procedure, 6 months, and 12 months for the presence of an ASD. With 2DE, iASDs were identified with color flow Doppler in the apical four-chamber or the subcostal view. If an iASD was detected, the larger color flow width at the level of the interatrial septum was recorded as the iASD diameter.

**Figure 1:**
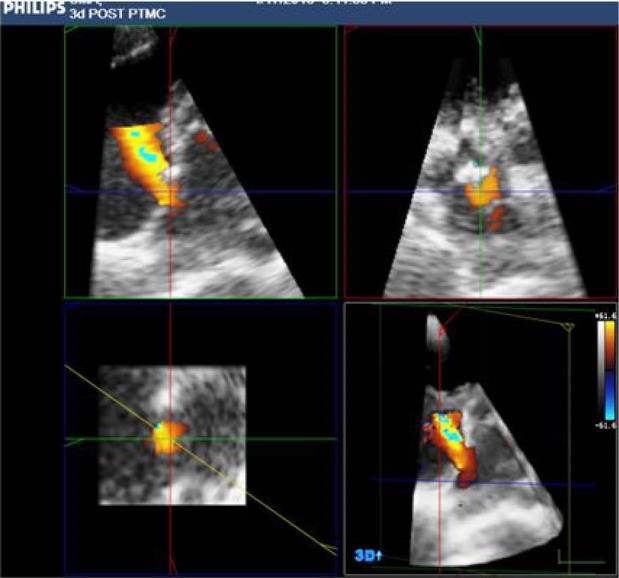
iSplice View of Post-processing of iASD by 3D Colour Datasets (RT3DE).

**Figure 2:**
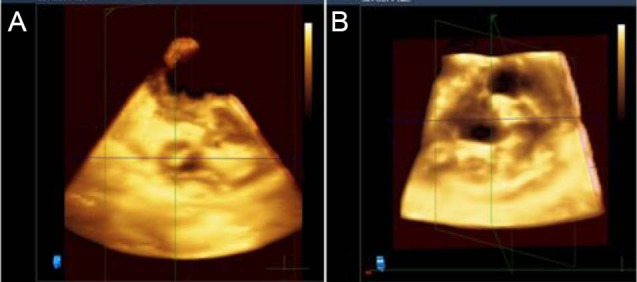
iASD detection after post-processing in a full volume RT3DE datasets. (A) iASD detection after post-processing in a full volume RT3DE datasets; (B) iASD evaluation by 3D Zoom (RT3DE).

RT3DE was performed with 2DE guidance, ECG gating or auto acquisition in atrial fibrillation patients. Datasets were acquired of live 3D, 3D zoom, full volume and 3Dcolour formats. The stored datasets were post processed in the same workstation with QLAB software for qualitative enhancement and quantification (Figure 1 and 2). RT3DE was also performed the day before and 24–48 hours after PTMC during the same examination as 2DE, using the same system and a matrix array transducer (X3-1, Philips). RT3DE images were obtained and stored. Method of data acquisition was based on recommendations of the Adhoc 3D Echo Protocol Working Group endorsed by the International Society of Cardiovascular Ultrasound.^14^ The recorded datasets were analyzed on a workstation using dedicated software.^15^

### Statistical Analysis

Quantitative variables were expressed as mean ± standard deviations and analyzed using unpaired *t* test and Pearson correlation. Agreement between the two methods of assessment was analyzed by the Passing and Bablok regression analysis. Intraclass correlation coefficient was assessed for the reliability analysis. Binary logistic regression was used for the analysis of influence of the study variables. Statistical significance was defined with *P*<.05. Microsoft Excel (2007), MedCalc version 15.2.2 and SPSS version 20.0 (IBM, Chicago, IL, USA.) was used for the organization and tabulation of results, and for statistical analysis.

## Results

### Baseline Characteristics of the Population

The mean age of the patients was 35.21 ± 10.26 years (13-61 years). Majority of the patients were in the age group of 20-40 years. Out of 110 patients, 36 (32.72%) were males and 74 (67.27%) were females. Mean height was 156.54 ± 9.47 cm (130 to 180 cm). Mean weight was 49.90 ± 10.78 kg (21 to 75 kg). Nine (8.10%) out of 110 study patients had previously undergone PTMC. Two (1.8%) of the 110 study population had undergone a previous surgical mitral commissurotomy. Eleven (10%) of the study population were in atrial fibrillation.

### Echocardiographic Parameters

The mean LV ejection fraction (EF) was 60.15 ± 5.75. Seven (6.4%) patients had mild LV dysfunction. Mean left atrial (LA) size was 45.55 ± 6.31 mm (33 to 65 mm). Mean right ventricular systolic pressure (RVSP) was 53.03 ± 24.53 mm Hg (16-130). The mean Wilkins score was 7.09 ± 1.21 (5 to 10). Mitral valve area (MVA) was assessed by planimetry, mean pre-procedure MVA was 0.82 ± 0.14 cm^2^ (0.5-1.2 cm^2^ ), mean post-procedure MVA was 1.75 ± 0.17 cm^2^ (range 1.22-2.00 cm^2^ ) and mean MVA change was 0.9 ± 0.19 cm^2^ ). The atrial septum was graded visually on TEE. Normal septum was seen in 12 (10.9%), thick septum in 11 (10%), while a majority had thin septum 82 (74.5%). Atrial septal aneurysm was noted in 5 (4.5%). Left atrial spontaneous echo contrast (LASEC) was assessed visually on TEE. Dense SEC was encountered in 51 (46.4%), mild SEC in 43 (39.1%), while no SEC was seen in 16 (14.5%) (Table 1).

**Table 1:** Demographic and Echocardiographic Parameters

**Characteristic**	**Patients**
Age (y)	
Mean ± SD	35.21 ± 10.26
Range	13-61
Sex	
Males	36 (32.72%)
Females	74 (67.27%)
Height (cm)	
Mean ± SD	156.54± 9.47
Range	130 to 180
Weight (kg)	
Mean ± SD	49.90 ± 10.78
Range	21 to 75
Previous PTMC	9 (8.10%)
Previous OMV/CMV	2 (1.8%)
Atrial Fibrillation	11 (10%)
LVEF (%)	
Mean±SD	60.15± 5.75
Range	42-70
LA size (mm)	
Mean ± SD	45.55 ± 6.31
Range	33 to 65
RVSP (mm)	
Mean±SD	53.03 ± 24.53
Range	16-130
Wilkins Score	
Mean±SD	7.09 ± 1.21
Range	5 to 10
TR Grade	
Trivial TR {1+}	25 (22.7%)
Mild TR {2+}	56 (50.9%)
Moderate TR {3+}	17 (15.5%)
Severe TR {4+}	12 (10.9%)

Abbreviations: SD, standard deviation; PTMC, percutaneous transvenous mitral commissurotomy; OMC, open mitral commissurotomy; CMC, closed mitral commissurotomy; LVEF, left ventricular ejection fraction; LA, left Atrium; RVSP, right ventricular systolic pressure; TR, tricuspid regurgitation.

### Catheterization Data

Regarding the PTMC procedure, the size of the balloon was selected according to the height. The larger balloon of size 25-28 was used in 90 (81.80%) cases, 24-28 in one patient (0.90%), 22-24 in one (0.90%) and the 21-24 size balloon was selected in 18 (16.40%) cases. The number of balloon inflations per procedure ranged from 1 to 3. The mean number of balloon inflations was 1.06 ± 0.28. The inflation pressure ranged from 22 to 26 mm, with mean inflation diameter of 24.24 ± 0.69. Invasive measurements with fluid filled catheters were obtained bean before and after the procedure. There was significant fall in mean LA from 20.65 ± 7.08 to 10.24 ± 4.54 mm Hg (*P *< .0001). There was significant fall in LA “a” wave peak from 23.74±13.34 to 13.33 ± 6.59 mm Hg (*P *< .0001) and “v” wave peak from 27.32 ± 9.08 to 14.75 ± 6.36 mm Hg (*P *< .0001). There was significant increase in left ventricular end diastolic pressure (LVEDP) from 5.60 ± 2.47 to 8.69 ± 3.25 mm Hg (*P *< .0001).

### RT3D Echo Comparison With 2D Echo

Pre-PTMC, none of the patients showed preexisting ASD on imaging by 2DE or RT3DE. Postprocedure, with color RT3DE an iASD was detected in 94 (85.5%) of patients while with standard transthoracic echocardiography (TTE), iASD was detected in 74 (67.3%) (*P *< .0001). Various modes of RT3DE and 2DE were analyzed for detection of iASD in the post procedure period. The proportional positive detection of iASD and statistical significance of usefulness of all modes of RT3DE are shown in Table 2. The most common site of iASD was in the fossa ovalis region in 44 (40%), followed by inferior vena cava (IVC) portion in 31 (28.2%), superior portion in 20 (18.2%) and in 15 (13.6%) the exact location could not be determined.

**Table 2:** iASD Detection by 2DE; 3D Color, 3D Zoom, 3D Full Volume and 3D Live

**Variable**	**iASD**		**Proportion (%)**
	**No**	**Yes**	
3D color	16	94	85.45
3D zoom	28	82	74.55
3D full volume	20	90	81.82
3D Live	22	88	80.00
2DE	36	74	67.27

Cochran’s Q test= 40.5333, n = 110, df = 4, *P* < .001

**Figure 3:**
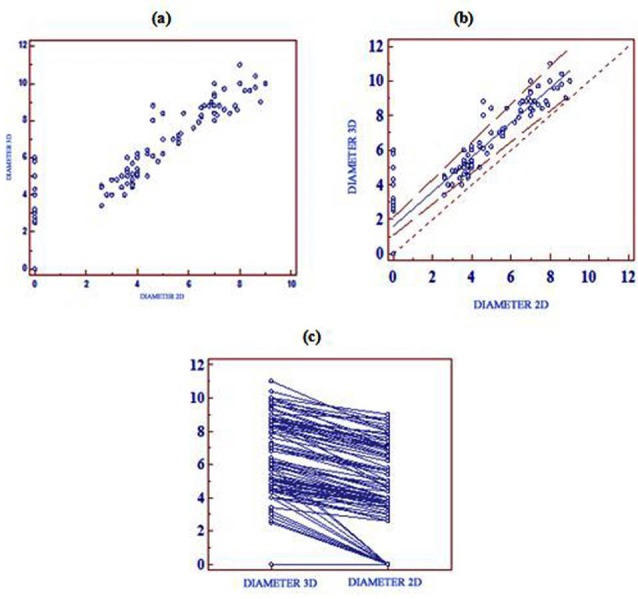
Scatter plot of iASD diameter by RT3DE and 2DE. (a) Scatter plot of iASD diameter by RT3DE and 2DE. (b) Passing and Bablok regression plot for iASD diameter by RT3DE and 2DE. (c) Connecting Dot-Plot of iASD diameter by RT3DE and 2DE.

The mean diameter of iASD measured by RT3DE was 5.41 ± 3.12 mm (range 2.5-11 mm), while 2DE measurements were smaller- mean diameter of 3.85 ± 2.83 mm (range 2-9 mm). On Pearson correlation, RT3DE is superior to 2DE for assessing the diameter of iASDs (r = 0.92; 95% CI for r = 0.88 to 0.94; *P *< .0001) (Figure 3a). It was possible to measure the area of iASD by RT3DE (mean 6.57 ± 3.81 mm^2^ , range 2.8-13.6 mm^2^ ), but could not be done directly by 2DE.

To measure the systematic, proportional and random differences between the two diagnostic methods of RT3DE and 2DE, Passing and Bablok regression method was used. Passing-Bablok compares two analytical methods, a test method against a reference/comparative method, to determine analytical accuracy. It showed that for measurement of iASD diameter, RT3DE did not have constant/proportional bias or any significant deviation from linearity (Table 3, Figure 3b). There was a linear relation between the measures of iASD diameter by RT3DE and 2DE (Figure 3c). As RT3DE was done by one and rarely two operators, reliabilility of repeated measures for consistency of results was analyzed. For RT3DE derived measurement of iASD diameter, the degree of consistency among measurements was significant (Table 4).

**Table 3:** Passing and Bablok regression iASD by RT3DE and 2D

** Regression Equation ** **(y = 1.600000 + 1.000000 x) **	
Systematic differences	
Intercept A	1.6000
95% CI	1.1000 to 2.1200
Proportional differences	
Slope B	1.0000
95% CI	0.9000 to 1.0909
Random differences	
Residual standard deviation (RSD)	0.7990
± 1.96 RSD Interval	-1.5661 to 1.5661
Linear model validity	
Cusum test for linearity	No significant deviation from linearity (P = .20)

**Table 4:** Analysis of the Reliability of Measurements by RT3DE

No. of Subjects (n)	110	
No. of raters (k)	2	
Model	The same raters for all subjects. Two-way model.	
Type	Consistency	
Measurements	DIAMETER RT3DE DIAMETER 2DE	
Intraclass correlation coefficient		
	Intraclass correlation^a^	95% CI
Single measures^b^	0.9183	0.8830 to 0.9433
Average measures^c^	0.9574	0.9379 to 0.9708

^a^ The degree of consistency among measurements; ^b^ Estimates the reliability of single ratings; ^c^ Estimates the reliability of averages of *k* ratings.

### Correlation of iASD With Clinical, Procedural and Echo Variables

The occurrence of iASD significantly and positively correlated with height of the patient, post-PTMC LVEDP, preprocedural and postprocedural LA ‘V’, pre- and post-PTMC LA mean (M), LA size, post-PTMC MVA, actual change in MVA and Wilkins score measurement (Table 5).

**Table 5:** iASD (RT3DE) correlation with demographic, Procedural (Cath) and echocardiographic parameters^a^

**Parameter**	**iASD**	**Mean**	***P *** ** Value**
Age (y, mean)	Yes	35.77	.19
	No	32.00	
Height (cm, mean)	Yes	157.34	.03^b^
	No	151.88	
Weight (kg, mean)	Yes	49.91	.98
	No	49.88	
EF (%)	Yes	60.33	.44
	No	59.13	
Pre-LVEDP (mm Hg)	Yes	5.70	.29
	No	5.00	
Post-LVEDP(mm Hg)	Yes	8.99	.01^b^
	No	6.94	
Pre-A (mm Hg)	Yes	23.95	.61
	No	22.56	
Post-A (mm Hg)	Yes	13.45	.67
	No	12.69	
Pre-V(mm Hg)	Yes	28.74	<.0001^b^
	No	18.94	
Post-V(mm Hg)	Yes	15.41	.007^b^
	No	10.81	
Pre-M (mm Hg)	Yes	21.55	.001^b^
	No	15.38	
Post-M (mm Hg)	Yes	10.66	.01^b^
	No	7.75	
Inflations (no.)	Yes	1.07	.32
	No	1.00	
Inflation pressure (mm Hg)	Yes	24.27	.27
	No	24.06	
LA (mm)	Yes	45.77	.03^b^
	No	42.25	
Pre-MVA (mm)	Yes	0.82	.62
	No	0.84	
Post-MVA (mm)	Yes	1.73	.01^b^
	No	1.85	
MVA Change (mm)	Yes	0.90	.06^b^
	No	1.00	
Wilkins score	Yes	7.27	.004^b^
	No	6.31	
RVSP (mm)	Yes	54.77	.07
	No	42.81	

^a^ Postprocedure; ^b^ Indicates a significant *P* value (*P *< .05)

Binary logistic regression of the demographic variables age, sex, height and weight revealed that only the patient height was influencing iASD (*P *= .04). Among the echocardiographic variables EF, LA size, pre- and post-MVA, Wilkins score and RVSP; only Wilkins score (*P *= .08) was significantly related to iASD. Invasive catheterization parameters of preprocedure and postprocedure LVEDP, LA ‘a’ wave, ‘v’ wave, mean pressure, PTMC balloon size, inflation diameter, number of inflations were analyzed. Pre-LVEDP (*P *= .02), post-LVEDP (*P *= .01), pre-V (*P *= .007) correlated significantly with the occurrence of iASD.

After the postprocedure RT3DE iASD evaluation, the study population was followed-up at 6 months and 1 year. While at the postprocedural period color 3DE was the most useful for detection of iASD, 3D full volume data sets were more useful at 6 months (60.9%) and 1 year (40.9%) follow-up. 2DE was uniformly less useful for iASD detection at follow-up periods (Table 6).

**Table 6:** iASD on Follow up by RT3DE and 2DE

**Variable**	**Postprocedure**	**6 months follow-up**	**1 year follow-up**
3D Color			
Yes	94 (85.5%)	61 (55.5%)	21 (19.09%)
No	16 (14.5%)	49 (44.5%)	89 (80.90%)
3D Full Volume			
Yes	90 (81.8%)	61 (55.5%)	21 (19.09%)
No	20 (18.2%)	49 (44.5%)	89 (80.90%)
3D Live			
Yes	88 (80%)	57 (51.8%)	16 (14.5%)
No	22 (20%)	53 (48.2)	94 (85.5%)
3D Zoom			
Yes	82 (74.5%)	49 (44.5%)	21 (19.1%)
No	28 (25.5%)	61 (55.5%)	89 (80.9%)
2D Echo			
Yes	74 (67.3%)	45 (40.9%)	11 (10%)
No	36 (32.7%)	65 (59.1)	99 (90%)

Abbreviations: iASD, iatrogenic atrial septal defect‏; RT3DE, real time three dimensional echocardiography; 2DE, two dimensional echocardiography.

### Subgroup Analysis

In 11 (10%) patients who had undergone previous PTMC, the mean iASD diameter (9.05 ± 0.92 vs 5.01 ± 3.01; *P *< .0001) and area (9.89 ± 2.96 vs 6.40±3.74; *P *= .004) were significantly higher than the naive patient population. On analysis of previous PTMC and iASD, there was a strong positive association (*P *= .03), but the association was of significance only at the 6 month follow up (*P *= .001) **(**Table 7**)**.

**Table 7:** Follow up of iASD in Previous PTMC Group

**iASD**	**Non-previous PTMC**	**Previous TMC**	**P Value**	**No AF**	**AF**	**P Value**
Postprocedure						
No	16 (14.5%)	0 (0%)	.16	16	0	.16
Yes	83 (75.5%)	11 (10%)		83	11	
6 months						
No	49 (44.5%)	0 (0%)	.01^a^	49	0	.02^a^
Yes	50 (45.5%)	11 (10%)		50	11	
1 year						
No	69 (62.7%)	5 (4.5%)	.10	72	2	<.0001^a^
Yes	30 (2.3%)	6 (5.5%)		27	9	

Abbreviations: AF, atrial fibrillation; PTMC; percutaneous transvenous mitral‏ commissurotomy‏. ^a^ Indicates a significant *P* value (*P *< .05)

In 11 (10%) patients with atrial fibrillation (AF), the mean diameter and area of iASD were higher than those in sinus rhythm. Of 11 (10%) patients with AF, all had iASD postprocedure. At 6 months follow up, all 11 (10%) had a persistent iASD. At 1 year follow up, only 9 (8.2%) had iASD visualized.

## Discussion

This is the first study to investigate iASDs after PTMC using RT3DE. The burden of chronic rheumatic heart disease is high in developing countries, with MS being the most common long-term expression of the disease. PTMC is a safe and effective procedure for relief of severe MS. PTMC requires transseptal access to the LA, which is created by puncture and dilatation of the interatrial septum by catheters. In view of widespread popularity of PTMC and many other forms of transseptal procedures requiring large-diameter transseptal catheters or sheaths, knowledge of the natural history of this inevitable complication is vital. This study assessed the use of RT3DE and incidence of iASD in a large cohort of patients undergoing transseptal catheterization during PTMC. RT3DE, once considered a research tool, is now an everyday clinical application.

### RT3DE Compared With Classic 2DE for Detection and Quantification of iASD

The atrial septum is one of the most difficult-to-visualize structures of the heart. An iASD after PTMC is variable in location and small. All the available diagnostic modalities come up short in evaluation of iASD, with reported incidences from 10% to 90%, depending on the technique used (Table 8).^3-11^ The traditional methods of iASD quantification have even more limitations. RT3DE eliminates the need for systematic imaging to detect iASD by using multiple techniques, some of which are semi-invasive or cumbersome.

**Table 8:** Comparison With Previous Studies

**Author/ Publication**	**Procedure/ TSCatheter Size**	**ASD Detection Method**	**No.**	**Results**
Yoshida et al^3^	PBMV; 14-Fr septal dilator	TEE, TTE, and oximetry	15	87% postprocedure iASDs day 1; 73% at 1 week, 47% at 1 month, 20% at 6 months by TEE
Ishikura et al^4^	PBMV; 14-Fr septal dilator	TTE	46	15.2% postprocedure iASDs on day 1; 6.5% at 3 months; 4.3% at 12 months; iASD directly correlated to longer procedure time
Cequier et al^5^	PBMV single (2 patients) or double balloon (66 patients)	Venovenous indicator dilution and oximetry	68	62% iASDs postprocedure; iASD present in 48% of 33 patients that had follow-up at 6 months (24% of initial 68 patients); iASDs associated with smaller LA, MV calcification, and smaller improvement in MV area
Casale et al^6^	PBMV single (n=27) or double balloon (n=123); 8-mm dilating atrial balloon,	Oximetry, TTE, and/or operative findings	150	19% iASDs at time of procedure; 41% of iASDs were persistent in those that had 10-month follow-up (6% of initial 150 patients); iASDs were associated with lower preprocedural cardiac output and presence of MV calcification
Hammerstingl et al.^7^	PVI/atrial fibrillation ablation; 8-Fr sheaths	TEE with Doppler or Contrast	42	19% iASDs at 9 months; iASD with RLS associated with preprocedure increased PAP and single TS puncture
Liu et al^8^	Diagnostic, 6 Fr; RFA, 7 Fr; PBMV, 14-Fr catheter	Oximetry, TTE, and TEE with color Doppler	176	All iASDS were after PBMV; iASD incidence 19% and 15% at 6 and 18 months, respectively for all TS procedures; iASDs associated with smaller MV area, higher transmitral gradient, and PAP
Rillig et al^9^	PVI/AF, double TS puncture; two 8-Fr sheaths	TEE with color Doppler or contrast	31	87% iASDs day 1; 3.7% at 3, 6, 12 months
Manjunath et al^10^	PTMC	TEE with color Doppler	209	Mean diameter of the iASD by TEE was 4.47 ± 1.7 mm; most common site of septal puncture was the inferior vena caval side; residual ASD was seen in 11 patients (8.7%) at 6-months
Smith et al^11^	MitraClip 22 F	TTE with color Doppler	52	Mean diameter was 6.0 ± 2.3 mm; At 6 months and 12 months, iASDs were identified in 27% significant difference in TR grade; persistent MR >2+ was more significant in the iASD group
Present study	PTMC	RT3DE 2DTTE	110	85% iASD post procedure, 56% at 6 month, 19% at 1yr follow up. Mean iASD diameter 5.41 ± 3.12 mm, area 6.57 ± 3.81mm^2^ iASD correlates with patient height, Wilkins score, pre-PTMC LA ‘v’, Post PTMC LVEDP. Persistence of iASD correlates with previous PTMC at 6 months, AF at 6 months and 1 yr RT3DE is more reliable and accurate for the study of iASD than 2DE

In our study, 2DE (TTE) detected iASD in 74 patients (67.27%) post-procedure, less than the detection rate of RT3DE, 94 patients (85.45%). 2DE was inferior to all the individual modes of RT3DE; this difference was statistically significant (*P *< .001). The mean diameter of iASD by 2DE was 3.85 ± 2.83 mm, significantly less than the 5.41 ± 3.12 mm by RT3DE (r = 0.92). By comparing the two analytical methods (2DE and RT3DE) by Passing and Bablok regression, any systematic (constant), proportional, and random bias was eliminated to yield the superiority of RT3DE quantitation. A more confident estimation and quantitation of iASD is possible by RT3DE because the entirety of the defect can be appreciated and planimetered by adjusting the imaging planes.

iASD size (diameter and area) was measured by one or two observers. Inter-method agreement was evaluated by means of the intraclass correlation coefficient. The analysis demonstrated a superior agreement when comparing RT3DE and 2DE; the intraclass correlation coefficient was 0.95 (CI: 0.93 to 0.97) and all individual measurements were positively correlated.

Our study suggests that RT3DE is more accurate than 2DE, particularly because it was more reproducible, correlated with 2DE, and was better for quantification. The lack of a gold standard for iASD measurements (even invasive methods are not sensitive enough for iASD detection) prevents accuracy assessment but the high rates of detection, reproducibility of results, feasibility of direct (en face) quantitation, and incremental value of various methods of RT3DE suggest that it is superior to 2DE and probably other techniques used for iASD assessment.

### The Incremental Value of RT3DE in Assessing iASD

Live 3D was usually the mode used first in RT3DE. iASD was detected in 88 patients (80%). Live 3D is usually the first mode used after 2D images. Though equal to other modes of RT3DE post-procedure, it was less accurate at 1-year follow-up. Live 3D’s advantages are dynamic imaging, not requiring gating, and immediate manipulation of datasets for image enhancement and cropping without the need for post-processing; its main drawback is a narrow sector, which can be overcome by lateral steer and other techniques.

3D zoom is similar to Live 3D in that a small area in the sector is selected; this provides better delineation of borders and surface rendering but can be limited by potential artifacts.

Because the color 3D technique incorporates both the color and grayscale images, it helps in the initial localization of the iASD. The still frame or dynamic images give an appreciation of the shape, relative location, and size of iASD relative to the atrial chambers. With this help in initial localization, subsequent studies (Full volume, 3D Zoom) were used for quantitation of iASDs. In our study, color 3D was by far the most sensitive, detecting iASD in 94 patients (85.5%) post-procedure. This trend was also appreciated during the follow-up, detecting iASDs in 61 patients (55.5%) at 6 months and in 21 patients (19.09%) at one year. Like the Live 3D, color 3D also employs a narrow sector, which was not a huge drawback for the study of atrial septum. Color bleeding and aliasing with regurgitant jets from mitral or tricuspid valve was rare; the main impediment in using 3D color clinically was that it required post-processing.

Full volume datasets are the mainstay of quantification with the QLAB software, providing various tools for image enhancement, cropping along 3 dimensions (X, Y, Z) or any plane, measurement of iASD in all three axes, and planimetry. The main drawbacks are storage issues, such as large datasets, requirement of gating, and the need for dedicated post-processing.

### Correlation of iASD With Clinical, Procedural, and Echo Variables

The occurrence of iASD in the postprocedural period significantly and positively correlated with height of the patient, post-PTMC LVEDP, preprocedural and postprocedural LA ‘V’, pre- and post-PTMC LA mean (M), LA size, post-PTMC MVA, actual change in MVA, and Wilkins score measurement. This probably reflects the anatomy of the atrial septum, severity and chronicity of MV disease, and LA remodeling affecting the procedure of PTMC, requiring more catheter manipulation, additional trauma to the atrial septum, and possibly suboptimal PTMC outcomes.

Logistic regression analysis of all the clinical, echo, and procedural parameters revealed that only patient height (*P* = .04), Wilkins score (*P* = .08), pre-PTMC LVEDP (*P* = .02), post-PTMC LVEDP (*P* = .01), and pre-PTMC ‘v’ (*P* = .007) correlated significantly with the occurrence of iASD.

Patients who have undergone previous PTMC tend to have more diseased valves; this translates into difficulty in manipulation of the catheters and suboptimal procedural outcomes, which may result in larger and possibly persistent iASDs. In our study, post-PTMC, the mean diameter of iASD was 9.05 ± 0.92 vs 5.01 ± 3.01 mm in this subgroup. On follow-up, the influence of previous PTMC was significant at 6 months and persistent in 6 out of 11 patients at 1 year.

The influence of AF on iASD was not studied in the post PTMC population in earlier studies. The iASD diameter was larger in AF patients; this difference was statistically significant (*P *< .0001). iASD area assessed by RT3DE was also higher in AF (9.8 ± 1.2 vs 6.84 ± 3.84) and the association was significant (*P *< .004). The persistence of iASD in AF was high at 1 year (82%, *P *< .0001). The apparent lack of serious adverse events like paradoxical embolism or stroke may be because all AF patients were on either antiplatelet and/ or anticoagulant medications.

### Clinical Perspective

There were no clinical adverse events like death, repeat PTMC procedure, paradoxical embolism, or transient ischemic attack (TIA)/stroke in the patients with iASD at up to 12 months of follow-up. Although only the clinical consequences of right-to-left shunting in patients with an iASD are frequently of concern, one cannot ignore the possible detrimental consequences of left-to-right cardiac shunting (right heart volume overload and pulmonary hypertension). In the absence of any clinically relevant adverse events, the need for specific therapy to be instituted or rigorous surveillance by various diagnostic techniques is not clear.

The presence and magnitude of any shunt will probably also be related to catheter size and the amount of trauma at the site of the atrial septal dilation. The procedural factors, such as balloon size, number of inflations, and inflation pressures, were not related to the occurrence of iASD. The factors associated with iASD were patient height, Wilkins score of MV, pre-PTMC LA ‘v’, and post-PTMC LVEDP, probably reflecting the influence of anatomical variations, more severely diseased valves, and higher LA pressures; these predict greater technical difficulty, resulting in additional trauma to the septum. Additionally, an optimal PTMC procedure that restores good hemodynamics may influence iASD.

Specific subgroups, like earlier mitral valvular interventions (previous PTMC) and AF, may be at risk of persistent iASD. Those with right-to-left shunting may benefit with judicial use of anti-platelet drugs or anticoagulants; further studies are warranted in this aspect.

RT3DE has proven value and superiority in the context of PTMC, for accurate Wilkins scores, MVA assessment, and detection of complications and is preferred especially in the post PTMC period, when other methods are inaccurate. Hence RT3DE should be integrated into routine echocardiographic examination and could replace traditional 2DE methods in relation to PTMC.

## Conclusion

RT3DE imaging is superior in accuracy to traditional 2DE techniques and can be used to quantify the iASD in the period after PTMC. All the modes of RT3DE are useful in the assessment of iASD; full volume datasets were more useful for quantitation. RT3DE is reliable and provides consistent results, irrespective of the operator. iASD measured by RT3DE correlates with several patient, procedural, and echocardiographic variables. RT3DE continues to be superior for follow-up evaluation of iASDs.

The incidence of iASD is high immediately after PTMC (85.5%); their detection depends on the type of imaging used, and there is a high rate of resolution of iASD, as only 19% have a residual iASD at one-year follow-up. iASDs occur most commonly in the fossa ovalis region; right-to-left shunt across an iASD is very rare. The persistence of iASD is higher in subgroups like those with a previous PTMC or AF. The strength of our study lies in it being one of the first applications of RT3DE for iASD after PTMC, with a systematic imaging protocol, large sample size, long follow-up period, statistical proof of the reliability and accuracy of results, and a clinical perspective on RT3DE and iASDs.

## Limitations

The ability to detect iASD after transseptal catheterization will depend on the attention given to the interatrial septum and the imaging modality used. We used transthoracic RT3DE alone, and this may underestimate the actual incidence of iASD. RT3DTEE, while the best option to visualize the atrial septum, was not used for the study, as it is semi-invasive and does not provide extra information on other aspects of post PTMC assessment besides adding to the cost. We could not determine the exact location of the puncture site—a possible contributing factor in the development of iASD—because the septostomy had been performed without the guidance of TEE. iASD morphology and rims were not analyzed in this study and shunt fractions were not calculated, RT3D contrast Echo was underutilized, and only two patients showed right-to-left shunts by the study. Although this study proves the reliability of RT3DE, it still needs dedicated post-processing and has a steep learning curve. Follow-up was for one year, which, though longer than many studies, may not have been adequate to study the natural history of these iASDs. The follow-up period may not have been adequate to document the rare complications, like paradoxical embolism with brain abscess and TIA/stroke or minor associations, like migraine.

## Ethics issues

This study was conducted with the approval of the Institutional ethics committee of Sri Venkateswara Institute of Medical Sciences (SVIMS). There was no economic burden on the participating patients. All investigations were performed as part of routine investigations. In accordance with the ethical guidelines of the 1975 Declaration of Helsinki, informed consent was obtained from each participant.

## Competing interests

None.
